# Management of Proximal Humerus Fractures in Elderly Patients: A Narrative Review

**DOI:** 10.7759/cureus.98292

**Published:** 2025-12-02

**Authors:** Ahmed Mohamed, Usman Fuad, Usama Farook, Ahmed Nagi

**Affiliations:** 1 Trauma and Orthopedics, Royal Cornwall Hospital, Truro, GBR; 2 Trauma and Orthopedics, Wirral University Teaching Hospital NHS Foundation Trust, Wirral, GBR; 3 Trauma and Orthopedics, University Hospitals Derby and Burton NHS Foundation Trust, Derby, GBR

**Keywords:** conservative management, elderly patients, open reduction internal fixation, profher trial, proximal humerus fractures, reverse shoulder arthroplasty

## Abstract

Proximal humerus fractures represent a significant healthcare burden in the elderly population. These injuries are the third most common fragility fractures, after hip and distal radius fractures. The management of proximal humerus fractures in elderly patients is controversial. Treatment options range from conservative management to surgical interventions. The choice depends on multiple factors, including fracture pattern, bone quality, patient functional demands, and comorbidities. This narrative review examines the current evidence regarding both conservative and operative management strategies for proximal humerus fractures in elderly patients. We discuss the classification systems, treatment algorithms, outcomes, and complications. The goal of this review is to provide clinicians with a comprehensive understanding of management options to optimize patient outcomes.

## Introduction and background

Proximal humerus fractures are common injuries in the elderly population. The incidence increases after the age of 60 years. Women are more frequently affected than men. This reflects the higher prevalence of osteoporosis among elderly women. The annual incidence ranges from 60 to 80 per 100,000 population. However, this rate increases to over 250 per 100,000 in patients over 80 years old [1].

Most proximal humerus fractures in elderly patients result from low-energy falls. The mechanism involves a fall onto an outstretched hand or directly onto the shoulder. Osteoporosis makes bones vulnerable to fractures, even with minor trauma. The proximal humerus is susceptible to fracture because of its anatomical structure. The transition from dense metaphyseal bone to weaker cancellous bone creates a stress point [2].

The management of these fractures has significantly evolved over the past decades. Traditional teaching favored conservative treatment for most fractures. Recent advances in surgical techniques and implants have expanded operative options. However, large-scale randomized trials have questioned whether surgery improves outcomes compared with conservative management. The debate continues regarding which patients benefit most from surgical intervention [3,4].

Patient-related factors significantly influence treatment decisions. Age, functional status, hand dominance, cognitive function, smoking, alcohol use, and comorbidities must be considered. Bone quality is critical when planning surgical fixation. Osteoporosis affects implant stability and healing potential. Additionally, elderly patients often have multiple medical conditions that increase surgical risks [5].

This review synthesizes the current evidence on managing proximal humerus fractures in elderly patients. We will examine both conservative and operative approaches. It is essential to understand the advantages and limitations of each treatment strategy. This knowledge helps clinicians make informed decisions tailored to individual patients’ needs.

## Review

Classification systems

Several classification systems exist for proximal humerus fractures. The Neer classification (Table 1) is the most widely used. It divides fractures based on the number of displaced fragments. A fragment is considered displaced if separated by more than 1 cm or angulated more than 45°. The four main fragments are the humeral head, greater tuberosity, lesser tuberosity, and the humeral shaft [6].

**Table 1:** Neer classification of proximal humerus fractures ORIF: Open Reduction Internal Fixation Source: [6]

Fracture type	Number of displaced fragments	Description	Typical management
One-part	0	No fragments displaced >1 cm or angulated >45°	Conservative
Two-part	1	One fragment displaced (surgical neck, greater tuberosity, lesser tuberosity, or anatomic neck)	Conservative or ORIF
Three-part	2	Two fragments are displaced, typically the surgical neck with either the greater or the lesser tuberosity	Conservative, ORIF, or arthroplasty
Four-part	3	Three fragments were displaced (surgical neck, greater tuberosity, and lesser tuberosity)	Arthroplasty or conservative
Fracture-dislocation	Variable	Any fracture pattern with glenohumeral dislocation	Reduction + fixation or arthroplasty
Head-splitting	Variable	Fracture line through the articular surface of the humeral head	Arthroplasty

The AO Foundation/Orthopedic Trauma Association classification provides a more detailed system (Table 2). Fractures are categorized into three main types: Type A fractures are extra-articular and unifocal. Type B fractures are extra-articular and bifocal. Type C fractures are intra-articular. Each type has further subdivisions based on specific fracture characteristics [7].

**Table 2:** AO/OTA classification of proximal humerus fractures Each type progresses in complexity and severity from subtypes 1-3, with corresponding worsening of prognosis AVN: avascular necrosis; AO/OTA: AO Foundation/Orthopedic Trauma Association Source: [7]

Type	Description	Fracture characteristics	Prognosis
Type A	Extra-articular, unifocal	Fracture does not involve the articular surface; single fracture line	Good
Subtype A1	Tuberosity fracture	Isolated greater or lesser tuberosity fracture	Good with anatomic reduction
Subtype A2	Impacted metaphyseal fracture	Surgical neck fracture with impaction	Fair; valgus impacted patterns have a better prognosis
Subtype A3	Nonimpacted metaphyseal fracture	Surgical neck fracture without impaction	Fair; higher risk of displacement
Type B	Extra-articular, bifocal	Two separate fracture lines; no articular involvement	Fair to poor
Subtype B1	With metaphyseal impaction	Surgical neck and tuberosity fracture with impaction	Fair
Subtype B2	Without metaphyseal impaction	Surgical neck and tuberosity fracture without impaction	Poor
Subtype B3	With glenohumeral dislocation	Bifocal fracture with shoulder dislocation	Poor; high risk of complications
Type C	Intra-articular	Fracture involves the articular surface of the humeral head	Poor
Subtype C1	Slight displacement	Articular fracture with minimal displacement	Fair with accurate reduction
Subtype C2	Marked displacement (impacted)	Articular fracture with significant displacement and impaction	Poor
Subtype C3	Marked displacement (dislocated)	Articular fracture with dislocation	Poor; highest risk of AVN

Despite their widespread use, classification systems have limitations. Interobserver reliability is often poor to moderate. Different surgeons may classify the same fracture differently. This variability affects treatment planning and outcome comparisons [7]. Three-dimensional CT imaging has improved the classification accuracy. However, agreement remains imperfect, even with advanced imaging [8].

The classification helps guide treatment decisions. Generally, minimally displaced fractures respond well to conservative treatment. Displaced two-part fractures may benefit from surgical fixation. Three-part and four-part fractures present greater challenges. These complex patterns often compromise the blood supply to the humeral head. The risk of avascular necrosis increases with fracture complexity [9].

Conservative management

Conservative management remains the primary treatment for proximal humeral fractures in elderly patients. Studies have shown that approximately 80% of these fractures are minimally displaced. These fractures heal well without surgery. The goal of conservative treatment is restoration of function and reduction of operative risks [10].

The initial phase focuses on pain control and protection. A collar-and-cuff sling provides comfortable support and immobilization while maintaining inline traction using the weight of the arm. Ice application helps to reduce swelling and pain during the first few days. Analgesics are prescribed based on the severity of pain. Elderly patients require careful medication management. Opioids should be used cautiously because of the risk of falls and cognitive effects. Early mobilization is crucial for achieving optimal outcomes. Pendulum exercises begin within the first week after injury. These gentle movements prevent stiffness without stressing the fracture. Passive range-of-motion exercises start around two weeks. The therapist moves the arm while the patient remains relaxed. This maintains joint mobility during healing. Active exercises gradually progress based on fracture healing. Radiographic union occurs between 6 and 12 weeks. Active-assisted exercises begin at approximately four to six weeks. The patient participates more actively in moving the shoulder. Strengthening exercises start after adequate bone healing. Complete recovery may take 6-12 months [11,12].

Several studies support conservative management for minimally displaced fractures. The landmark Proximal Fracture of the Humerus: Evaluation by Randomization (PROFHER) trial conducted by Rangan et al. represents the largest randomized controlled trial comparing surgery with conservative treatment [3,4]. This pragmatic multicenter study recruited 250 patients aged ≥16 years from 32 hospitals in the United Kingdom. The trial included patients with displaced fractures involving the surgical neck, including two-part, three-part, and four-part fractures. At two-year follow-up, the trial found no significant difference in functional outcomes between surgical and nonsurgical groups. The surgical group had more complications, and subsequent surgery rates were similar between groups. The economic evaluation after five years found that surgery was not cost-effective [13]. The PROFHER trial provides strong evidence supporting conservative management of most displaced proximal humerus fractures.

Olerud et al. compared conservative treatment with locked plating for displaced three-part fractures in elderly patients. At two years, no significant difference existed in patient-reported outcomes. The conservative group had fewer complications. These findings suggest that many displaced fractures heal adequately without surgery [14].

However, conservative treatment has limitations. Some fractures remain unstable and displace further. Malunion can result in a reduced range of motion. Severely displaced greater tuberosity fractures may cause subacromial impingement. Careful patient selection is essential. Close radiographic monitoring ensures early detection of unacceptable displacements [15].

Patient compliance affects conservative treatment success. Elderly patients may struggle with exercise protocols. Cognitive impairment can limit therapy participation. Social support and living situation influence recovery. Home therapy may be necessary for patients with mobility limitations. Regular follow-up appointments monitor progress and adjust treatment plans [16].

Surgical management: general considerations

Surgical intervention is considered for specific fracture patterns and patient populations. The decision depends on fracture characteristics, patient factors, and surgeon experience. There are a few absolute indications for surgery. These include open fractures, vascular injury, head split fractures, and irreducible fracture-dislocations [17]. Relative indications are more common and controversial. Displaced two-part surgical neck fractures may benefit from fixation in young and active patients, whereas three-part and four-part fractures often require surgery. However, outcomes remain unpredictable, even with optimal surgical techniques [18].

Several surgical options exist for fixation. The choice depends on fracture pattern and bone quality. Closed reduction and percutaneous pinning are minimally invasive. Open reduction and internal fixation (ORIF) using plates provides a stable fixation. Intramedullary nailing is another fixation option. Hemiarthroplasty or reverse total shoulder arthroplasty (RTSA) addresses unreconstructible fractures [19].

Percutaneous Pinning

Percutaneous pinning is a minimally invasive surgical technique. The procedure begins with closed reduction under fluoroscopic guidance. Traction and manipulation restore the anatomical alignment. Kirschner wires are then inserted percutaneously. Common entry points include the greater tuberosity and the lateral deltoid area. Pins are advanced into the humeral head and medial cortex. Multiple pins in different planes provide three-dimensional stability. This technique works best for two-part fractures with good bone quality [20]. The advantages of this technique include minimal soft tissue dissection. Blood supply to fracture fragments is preserved. Surgical time is shorter than that of open procedures. Postoperative pain is generally lower. Hospitalization was minimal. This technique is more cost-effective than other surgical options [21]. Complications include pin migration, which causes soft tissue irritation and later displacement at the fracture site, pin tract infection, and inadequate reduction. Careful pin placement and an adequate number and length prevent migration and displacement. The pins are removed after four to six weeks. This allows early healing while preventing long-term complications [20,21].

Outcomes depend heavily on fracture pattern and bone quality. Simple two-part fractures achieve good results. Complex fractures are less suitable for this technique. Loss of reduction can occur if fixation is inadequate. Studies show acceptable functional outcomes in selected patients. Desai et al. reported good outcomes using percutaneous pinning for two-part and three-part fractures. However, they noted higher failure rates in osteoporotic bone. The technique requires significant fluoroscopy time. Radiation exposure to patients and surgeons is a concern. Proper technique and experience minimize this risk [22-24].

Open Reduction and Internal Fixation

ORIF using locking plates is common. This technique provides stable fixation for displaced fractures. Locked screws create fixed-angle constructs. This improves stability in osteoporotic bone compared to conventional plates [25].

The deltopectoral approach is most commonly used. This interval preserves the axillary nerve. The anterior circumflex vessels are identified and protected. The fracture is exposed and reduced under direct visualization. Sutures or cerclage wires can be used for fragment control [26]. The locking plate is positioned on the lateral aspect of the proximal humerus. Proper plate placement is critical for optimal outcomes. The plate should be positioned below the greater tuberosity. This prevents subacromial impingement. Locking screws are inserted into the humeral head. Multiple screws in different directions provide secure fixation [27]. Tuberosity repair is crucial for rotator cuff function. The greater and lesser tuberosities should be anatomically reduced. Sutures through the rotator cuff tendons reinforce fixation. These sutures are tied around or through the plate. Secure tuberosity fixation prevents secondary displacement [28].

Several studies have evaluated ORIF outcomes. Results are variable and depend on multiple factors. Bone quality significantly affects fixation stability. Fjalestad et al. compared ORIF with conservative treatment in a randomized trial of elderly patients with displaced three-part and four-part fractures [15]. They found no significant difference in functional scores at two years. The surgical group had more complications. Shrestha et al. included 60 patients with proximal humerus fractures in their prospective study, which showed the effectiveness of ORIF using a Proximal Humeral Internal Locking System plate in achieving anatomical reduction and functional restoration [29]. Therefore, patient selection for this approach is crucial for satisfactory outcomes and complications avoidance.

Complications include infection, nerve injury, and hardware prominence. The axillary nerve is at risk during surgery. Careful dissection and retractor placement protect the nerve. Subacromial impingement from proud hardware causes pain. Revision surgery for hardware removal may be necessary. Primary varus collapse is a common complication in osteoporotic bone. Screw cutout through the humeral head occurs when fixation fails [30].

Intramedullary Nailing

Intramedullary nailing is an alternative fixation method. A nail is inserted into the medullary canal from the greater tuberosity. Proximal locking screws stabilize the humeral head. Distal locking screws prevent nail migration and rotation. Advantages include minimal soft tissue stripping. The blood supply to bone fragments is preserved. The nail provides load sharing rather than load bearing. This may reduce fixation failure in weak bone. The technique is less demanding than plate fixation [31]. A proper nail insertion point is critical. The entry point is at the greater tuberosity apex. An incorrect entry causes a tuberosity fracture or a rotator cuff injury. The nail should be buried below the bone surface. Prominent hardware causes subacromial impingement and pain [32].

The outcomes of intramedullary nailing compared to locking plate fixation depend on multiple factors. Boadi et al. reviewed indications and technical considerations for both techniques in a comprehensive analysis. They noted that intramedullary nailing may be preferred for two-part surgical neck fractures with good bone quality and minimal comminution. The technique offers the advantages of shorter operative time and less soft tissue disruption. However, locking plates are generally preferred for fractures involving the tuberosities or with significant metaphyseal comminution. Both techniques struggle with osteoporotic bone. Patient age, fracture pattern, and bone quality are critical factors in selecting the appropriate fixation method [33].

Whiting et al. compared the operative outcomes of two-part and three-part proximal humerus fractures treated with either intramedullary nailing or open reduction internal fixation. Their study included patients with and without ipsilateral shaft fractures and head split patterns. The results showed comparable functional outcomes between the intramedullary nailing and ORIF groups. Complication rates were similar between techniques. However, the choice of fixation was influenced by fracture complexity and associated injuries. Patients with concomitant shaft fractures may benefit from intramedullary fixation that spans both fracture sites [34].

Complications with intramedullary nailing include proximal screw cutout in osteoporotic bone. Malunion can occur in varus. Tuberosity displacement compromises rotator cuff function. Proper nail insertion and screw placement techniques are essential to minimize complications. Contraindications include severe osteoporosis and metaphyseal comminution. The nail requires adequate bone stock for proximal screw fixation. Complex fractures with tuberosity involvement are challenging. Some surgeons prefer alternative techniques for these patterns [33,34].

Hemiarthroplasty

Hemiarthroplasty involves replacing the humeral head with a prosthetic implant. This option is considered for unreconstructable fractures. Four-part fractures in elderly patients are common indications. Head-split fractures with articular surface damage also qualify. The procedure involves removing the fractured humeral head. The shaft is reamed to accommodate the prosthetic stem. Proper version and height are crucial for function. The prosthesis is cemented or press-fit, depending on bone quality [35,36].

Tuberosity repair is the most important technical aspect. The greater and lesser tuberosities must be secured anatomically. Sutures through the rotator cuff tendons are anchored to the prosthesis. Tuberosity healing determines the functional outcomes. Tuberosity union failure results in poor function [37].

The outcomes of hemiarthroplasty are variable and depend on multiple factors. Kontakis et al. performed a systematic review of the early management of proximal humeral fractures with hemiarthroplasty [38]. Their analysis included 16 studies with 810 patients. The mean Constant score was 56.6 out of 100. The mean forward elevation ranged from 101° to 105.7°, external rotation from 18° to 30.4°, and abduction averaged 92.4°. Pain relief was generally reliable in 73%-93% of patients. However, functional recovery was often limited, with many patients achieving only a moderate range of motion. The systematic review found that tuberosity healing was the most important factor determining outcomes. Patients with anatomically healed tuberosities achieved significantly better functional results than those with tuberosity complications [38].

More recent evidence from Nguyen Manh et al. evaluated the outcomes of shoulder hemiarthroplasty for complex proximal humeral fractures in 32 patients [39]. At a mean follow-up of 18 months, the mean Constant score was 62.5 points. Forward flexion averaged 115°, abduction 108°, and external rotation 28°. Pain relief was achieved in 87.5% of patients. Tuberosity healing occurred in 68.7% of cases. Patients with healed tuberosities had significantly better functional outcomes than those with nonunion or malunion. The complications included tuberosity nonunion (31.3%), shoulder stiffness (15.6%), and infection (3.1%). The study confirmed that tuberosity healing is critical for active range of motion and functional recovery. Technical factors, including proper prosthetic height, correct version, and secure tuberosity fixation, are essential for optimal outcomes [39].

Complications include tuberosity nonunion, instability, and stiffness. Infection is a concern with any arthroplasty. Nerve injury can occur during surgery. Aseptic loosening may develop over time. Revision surgery for failed hemiarthroplasty is challenging [40].

Reverse Total Shoulder Arthroplasty

RTSA has revolutionized fracture treatment in elderly patients. This prosthesis reverses normal shoulder anatomy. The ball attaches to the scapula, and the socket connects to the humerus. This design does not rely on the rotator cuff for function. RTSA offers several advantages for fracture treatment. Tuberosity healing is less critical for outcomes. The deltoid muscle elevates the arm without the rotator cuff. This benefits elderly patients with chronic rotator cuff tears. Pain relief is reliable. Functional outcomes exceed those of hemiarthroplasty [41,42].

Indications for RTSA include complex fractures in elderly patients. Four-part fractures in patients over 70 years are appropriate. Preexisting rotator cuff pathology favors RTSA. Revision cases after failed fixation or hemiarthroplasty are suitable [43].

Several studies have compared RTSA with other fracture treatments. Sebastiá-Forcada et al. conducted a randomized controlled trial comparing RTSA with hemiarthroplasty in 62 patients older than 70 years. At a mean follow-up of 28.5 months, patients who underwent RTSA had significantly higher University of California, Los Angeles, scores (29.1 vs. 21.1; p = 0.001) and Constant scores (56.1 vs. 40.0; p = 0.001). Forward elevation was significantly better in the RTSA group (120° vs. 80°; p = 0.001), as was abduction (113° vs. 79°; p = 0.001). The tuberosity union rate was higher with RTSA (64% vs. 56%), and importantly, the lack of tuberosity union did not compromise outcomes in patients who underwent RTSA but did significantly affect the results of hemiarthroplasty [44].

Gallinet et al. performed a systematic review and meta-analysis comparing RTSA and hemiarthroplasty. Their analysis included multiple studies and found that RTSA patients had significantly better clinical outcome scores, forward flexion, and abduction than those undergoing hemiarthroplasty. External rotation was similar between the groups. The complication rate was higher in the RTSA group, but the reoperation rate was lower. The revision rate was higher in hemiarthroplasty patients. These findings support RTSA as the preferred arthroplasty option for complex proximal humerus fractures in elderly patients [45].

Long-term outcomes after fracture RTSA are encouraging. Gallinet et al. reported outcomes in 422 patients from a multicenter study with a mean follow-up of over three years [45]. Patients maintained good function over time, with mean Constant scores of 61 points. Pain relief was excellent and sustained. Revision rates remained low at long-term follow-up [46].

Tuberosity healing after RTSA is correlated with better outcomes. Jain et al. performed a systematic review and meta-analysis to examine this relationship. They found that patients with healed tuberosities had significantly better forward flexion, external rotation, and functional scores than those with nonhealed tuberosities. However, even patients without tuberosity healing achieved acceptable functional outcomes, unlike hemiarthroplasty, where tuberosity healing is critical [47].

Complications can occur with RTSA. Instability can develop if soft tissue tension is inadequate. Infection is a serious complication requiring implant removal. Scapular notching is visible on radiographs in many patients but is often asymptomatic. Technical factors affect RTSA outcomes. Proper component positioning is critical. Glenoid baseplate fixation must be secure. Experience with the RTSA technique improves results [48,49].

Treatment algorithm

Developing a treatment algorithm requires considering multiple factors. The fracture pattern is the primary consideration. Patient age, functional demands, and comorbidities also influence decisions. Bone quality and surgeon experience affect surgical success.

Minimally displaced one-part fractures are treated conservatively. These fractures are stable and heal reliably. Early mobilization protocols optimize outcomes. Close radiographic follow-up ensures stability [10].

Displaced two-part surgical neck fractures present treatment challenges. The PROFHER trial demonstrated that surgery does not provide any advantage in the treatment of these fractures. Conservative treatment produces comparable outcomes in most patients. However, young and active elderly patients may benefit from fixation in selected cases. Shared decision-making with patients is important [3,4].

Greater tuberosity fractures with >5 mm displacement often require surgery. Fixation prevents subacromial impingement. ORIF using suture anchors or screws provides stable fixation. Tuberosity healing reliably restores the rotator cuff function [50].

Three-part fractures are challenging. The PROFHER trial included these fractures and found no benefit of surgery [3,4]. Conservative treatment may produce acceptable results. ORIF is an option in selected patients with good bone quality. The decision depends on patient factors and the surgeon’s experience [25].

Four-part fractures in elderly patients traditionally require arthroplasty. However, the PROFHER trial challenged this approach by including four-part fractures and demonstrating acceptable outcomes with conservative treatment [3,4]. ORIF has a high failure rate in osteoporotic bones [51]. When arthroplasty is chosen, RTSA provides superior functional outcomes compared to hemiarthroplasty in patients aged >70 years [45,46].

Fracture-dislocations require urgent reduction. After reduction, the treatment follows the guidelines for the fracture pattern. Surgical stabilization is often required. Anatomic restoration is challenging. Outcomes are generally worse than fractures without dislocation [52].

Head-splitting fractures damage the articular surface. Arthroplasty is usually necessary. RTSA provides better outcomes than hemiarthroplasty. Conservative treatment is inappropriate for these injuries [53].

Patient preferences should guide treatment decisions. Some elderly patients prioritize avoiding surgery. Others prefer aggressive treatment for better function. Frank discussions about realistic expectations are essential. Shared decision-making respects patient autonomy [54].

## Conclusions

Proximal humerus fractures in elderly patients present significant treatment challenges. Both conservative and operative management have roles depending on patient and fracture characteristics. Conservative treatment is appropriate for most minimally displaced fractures. Functional outcomes are acceptable for elderly patients with modest demands. Surgical intervention with ORIF has specific indications and patient selection is critical for its success. RTSA has emerged as the preferred surgical option for complex fractures. Superior functional outcomes compared to hemiarthroplasty are well-documented. Elderly patients with unreconstructable fractures benefit the most from this technique. However, conservative treatment remains a reasonable alternative, even for complex fractures.

Future research should identify the patients who benefit from surgery. Current evidence shows no clear advantage for most fractures. A better understanding of predictive factors would improve decision-making. New technologies and techniques require rigorous evaluation before widespread adoption.
